# Effects of cLFchimera peptide on intestinal morphology, integrity, microbiota, and immune cells in broiler chickens challenged with necrotic enteritis

**DOI:** 10.1038/s41598-020-74754-x

**Published:** 2020-10-19

**Authors:** Ali Daneshmand, Hassan Kermanshahi, Mohammad Hadi Sekhavati, Ali Javadmanesh, Monireh Ahmadian, Marzieh Alizadeh, Ahmed Aldawoodi

**Affiliations:** Department of Animal Science, Faculty of Agriculture, Ferdowsi University of Mashhad, Mashhad, Iran

**Keywords:** Infection, Bacterial infection, Parasitic infection

## Abstract

Three hundred and sixty 1-day-old male broiler chicks were randomly allocated to 4 treatments of 6 replicates to evaluate the effects of cLFchimera, a recombinant antimicrobial peptide (AMP), on gut health attributes of broiler chickens under necrotic enteritis (NE) challenge. Treatments were as follows: (T1) unchallenged group fed with corn-soybean meal (CSM) without NE challenge and additives (NC); (T2) group fed with CSM and challenged with NE without any additives (PC); (T3) PC group supplemented with 20 mg cLFchimera/kg diet (AMP); (T4) PC group supplemented with 45 mg antibiotic (bacitracin methylene disalicylate)/kg diet (antibiotic). Birds were sampled for villi morphology, ileal microbiota, and jejunal gene expression of cytokines, tight junctions proteins, and mucin. Results showed that AMP ameliorated NE-related intestinal lesions, reduced mortality, and rehabilitated jejunal villi morphology in NE challenged birds. While the antibiotic non-selectively reduced the count of bacteria, AMP restored microflora balance in the ileum of challenged birds. cLFchimera regulated the expression of cytokines, junctional proteins, and mucin transcripts in the jejunum of NE challenged birds. In conclusion, cLFchimera can be a reliable candidate to substitute growth promoter antibiotics, while more research is required to unveil the exact mode of action of this synthetic peptide.

## Introduction

Necrotic enteritis (NE) is well-known as a detrimental disease in the poultry industry which results in production losses, increased mortality, reduced welfare of birds, and also increased risk of contamination of poultry products for human consumption^1^. The etiologic cause of NE is *Clostridium perfringens* (*C. perfringens*), a spore-forming Gram-positive bacterium that naturally inhabits the gastrointestinal tract of farm animals^2^.

Antibiotics have been widely used to control NE in poultry farms, while the administration of growth-promoting antibiotics is extensively forbidden due to the rapid spread of the antibiotic resistance in human cases^3^. This prohibition of antibiotic use in the livestock industry has inspired researcher into safe substitutions for antibiotics. Consequently, several additives have been introduced to the market, such as pro and prebiotics, essential oils, acidifiers, and antimicrobial peptides^4^.

Antimicrobial peptides (AMPs) are endo-exogenous polypeptides comprised of less than 50 amino acids, characterized by cationic amphipathic properties, and produced by host defense systems or synthetically supplied to the diet in order to protect a host from pathogenic microbes^5,6^. These peptides show broad-spectrum antimicrobial activities against various microorganisms, including Gram-positive and Gram-negative bacteria, fungi, and viruses^7^; they are well-known for their roles as competent alternatives for antibiotics in farm animal production^8–11^. Previous studies have demonstrated that AMPs could improve growth performance, nutrient digestibility and gut health, positively alter intestinal microbiota, and enhance immune function in pigs and broilers.

The AMP, cLFchimera, is a heterodimeric peptide designed to mimic two antimicrobial domains, Lactoferricin (LFcin) and Lactoferrampin (LFampin), which are present in the N1-domain of camel lactoferrin (cLF)^12^. More recently, the recombinant form of cLFchimera has been cloned and expressed in *E. coli*^12^ and *L. lactic*^13^. The results of in vitro studies showed that cLF36 has antibacterial^12,13^ antiviral^14^, and anticancer^15^ properties. Further, a previous in vivo study asserted that supplementing *E. coli* challenged broilers with cLFchimera improved jejunal villi morphology, restored microbial balance in the ileum, and improved gene expression of cytokines and tight junctions in the jejunum of challenged broiler chickens^16^. Therefore, the objective of this study was to evaluate the effectiveness of cLFchimera as an alternative to growth enhancer antibiotics on intestinal morphology, microflora, and gene expression of immune cells and junctional proteins in broiler chickens challenged with NE, as an animal model for infectious disease.

## Materials and methods

All experimental protocols involving animals in the present study were approved by Institutional Animal Care and Use Committee of Ferdowsi University of Mashhad (Protocol number 3/42449) and performed in accordance with relevant guidelines and regulations to minimize animal pain, suffering, and distress.

### Birds, treatments, and experimental design

A total of 360 1-day-old male chicks (Cobb 500) were purchased from a local hatchery, weighed and randomly assigned to 4 treatments with six replicates containing 15 birds in each replicate. Treatments were as follow: 1) negative control birds received a corn-soybean meal basal diet without AMPs, antibiotic, and NE challenge (NC); 2) positive control birds challenged with NE (PC); 3) PC birds challenged with NE and supplemented with 20 mg peptide/kg diet (AMP); 4) PC birds challenged with NE and supplemented with 45 mg antibiotic (bacitracin methylene disalicylate)/kg diet (antibiotic). All diets were in mash form and formulated to meet or exceed the minimum requirements of Cobb 500 (Table 1). Feed and water and were provided ad libitum. Chicks were reared in floor pens (1.1 m × 1.3 m) covered with wood shavings. Temperature and lighting programs were adjusted based on the guidelines of the Cobb 500 strain.

**Table 1:** Composition of experimental diets.

Ingredient (%)^1^	Starter (0–10 days)	Grower (11–22 days)
Corn	56.81	58.16
Soybean meal (44.0%)	36.01	34.80
Soybean oil	3.18	3.40
Dicalcium phosphate	1.79	1.65
Limestone	0.97	0.93
Salt	0.35	0.30
Mineral-vitamin premix^2^	0.50	0.50
DL-Methionine	0.17	0.15
L-Lysine HCl	0.22	0.12
**Calculated nutrients**		
AME (kcal/kg)	3000.00	3080.00
Crude protein (%)	21.00	19.00
Calcium (%)	0.90	0.84
Available phosphorus (%)	0.45	0.42
Sodium (%)	0.16	0.16
Methionine (%)	0.50	0.47
Methionine + cystene (%)	0.98	0.86
Lysine (%)	1.32	1.18

^1^Antibiotic (45 mg bacitracin methylene disalicylate/kg diet) and peptide (20 mg/kg diet) were added on top and thoroughly mixed.

^2^Added per kg of feed: vitamin A, 7,500 UI; vitamin D3 2100 UI; vitamin E, 280 UI; vitamin K3, 2 mg; thiamine, 2 mg; riboflavin, 6 mg; pyridoxine, 2.5 mg; cyanocobalamin, 0.012 mg, pantothenic acid, 15 mg; niacin, 35 mg; folic acid, 1 mg; biotin, 0.08 mg; iron, 40 mg; zinc, 80 mg; manganese, 80 mg; copper, 10 mg; iodine, 0.7 mg; selenium, 0.3 mg.

### AMP production

cLF chimera was derived from camel lactoferrin (cLF) consisting of 42 amino acids and has primary sequence of DLIWKLLVKAQEKFGRGKPSKRVKKMRRQWQACKSSHHHHHH. In addition, the results of our previous study showed that cLFchimera had antioxidant activity (IC50: 310 μ/ml) and its activity was not affected after 40 min of boiling^17^ (for more details regarding production process, please review previous papers^12,13,17^). Briefly, preparation of recombinant plasmid vector was conducted through transforming recombinant expression vector harboring synthetic cLFchimera into DH5α bacterium^12,13^. Next, the latter bacterial colonies were cultured to harvest plasmid extraction. The recombinant vector was then transferred into *E. coli* BL21 (DE3) as an expression host and cultured in 2 ml Luria–Bertani broth (LB) medium for overnight according to standard protocol^18^. In the next step, cultured materials were inoculated in 50 ml LB and incubated at 37 °C with shaking at 200 rpm. Then, isopropyl-β-d-thiogalactopyranoside (IPTG) was added to a final concentration of 1 mM and incubated at 37 °C for 6 h after IPTG induction. Periplasmic protein was collected at different times after IPTG induction (2, 4, and 6 h) according to the method described by de Souza Cândido et al.^19^ and analyzed on 12% SDS-PAGE. To purify expressed peptide, Ni–NTA agarose column was used based on manufacturer's instruction (Thermo, USA). The quality of purified recombinant peptide was assessed on a 12% SDS-PAGE gel electrophoresis, while the Bradford method was used to analyze the quantity of recombinant peptide. More recently, an *E. coli* expression system was developed in our laboratory that is able to produce 0.42 g/L of recombinant peptide. Finally, four grams of peptide previously obtained from the recombinant *E. coli* were purified, lyophilized, and thoroughly mixed with 1 kg soybean meal and then supplemented to the relevant experimental diets.

### NE challenge

A previously described method of inducing NE was used with some modifications^20^. Briefly, on day 16, chicks in NC group were administered a single 1 ml oral dose of sterile phosphate-buffered saline (PBS) (uninfected) as a sham control, while PC, peptide and the antibiotic groups were orally inoculated with 5,000 attenuated vaccine strain sporulated oocysts each of *E. maxima*, *E. acervulina,* and *E. tenella* (Livacox T, Biopharm Co., Prague, Czech Republic) in 1 mL of 1% (w/v) sterile saline. On days 20 and 21, birds in the NE groups were orally inoculated with 1 ml of broth containing *C. perfringens* isolated from broilers meat^21^, CIP (60.61) containing 10^7^ cfu/ml in thioglycollate (Thermo-Fisher Scientific Oxoid Ltd, Basingstoke, UK) supplemented with peptone and starch. Inoculated *C. perfringens* was analysed using PCR to confirm the presence of *netB* gene required for inducing NE in broilers according to Razmyar et al.^22^. The unchallenged group received the same dose of sterilized broth.

### Growth performance

On days 10 and 22, average daily gain (ADG), average daily feed intake (ADFI) and feed conversion ratio (FCR) were calculated using body weight (BW) and the weight of feed which remained in each pen. The ADG, ADFI and FCR were calculated over the specific and comprehensive periods of the study (0–10, 11–22, and 0–22 days). The feed conversion ratio for each period was readjusted based on the mortality data per pen per day, if any.

### Sample collection and lesion score

On days 10 and 22, two birds from each pen (12 birds/treatment) were randomly selected for euthanasia by cervical dislocation. The viscera was excised from the specimens, the intestine discretely separated and the adherent materials were precisely removed. The ileum was gently pressed to aseptically collect ileal content into sterile tubes for microbiological analysis. A section of approximately 5 cm from mid-jejunal tissues was meticulously separated for morphological analysis. A 2 cm section from the mid-jejunum was detached, rinsed in cold phosphate-buffered saline (PBS), immediately immersed in RNAlater (Qiagen, Germantown, MD), and stored at − 20 °C for subsequent gene expression determination. On day 22, NE lesions of duodenum, jejunum, and ileum from 2 birds per pen were scored on a scale of 0 (none) to 6 (high) as described previously^23^.

### Intestinal morphology

The method used to prepare samples for morphometry analysis was previously described by Daneshmand et al.^24^. Briefly, jejunal samples were stored in a 10% formaldehyde phosphate buffer for 48 h. The samples were then processed on a tissue processor (Excelsior AS, Thermo Fisher Scientific, Loughborough, UK), fixed in paraffin using an embedder (Thermo Fisher Histo Star Embedder, Loughborough, UK), and cut with a microtome (Leica HI1210, Leica Microsystems Ltd., Wetzlar, Germany) to a length of 3 cm per slice. The slices were placed on a slide and dehydrated on a hotplate (Leica ASP300S, Leica Microsystems Ltd., Wetzlar, Germany) and dyed with hematoxylin and eosin. Finally, the dyed slices of jejunal were examined under a microscope (Olympus BX41, Olympus Corporation, Tokyo, Japan). A total of 8 slides were prepared from the jejunal segment per bird, and ten individual well-oriented villi were measured per slide (80 villi/bird). The average of each measurement per sample was reported as the respective a mean for each bird. Villus width (VW) was measured at the base of each villus; villus height (VH) from the top of the villus to the villus-crypt junction, crypt depth (CD) from the base of the adjacent villus to the sub-mucosa, the ratio of VH/CD and villus surface area were calculated.

### Microbial count

The methods used to count the populations of *E. coli*, *Clostridium *spp., *Lactobacillus *spp., and *Bifidobacterium *spp*.* in the ileal content were described elsewhere^25^. In summary, the ileal contents of a sample were thoroughly mixed, serially diluted tenfold from 10^−1^ to 10^−7^ with sterile PBS, and homogenized for 3 min. Next, dilutions were plated on different agar mediums. Regarding the enumeration of bacteria, *Lactobacillus *spp*.* and *Clostridium *spp*.* dilutions were plated on MRS agar (Difco, Laboratories, Detroit, MI) and SPS agar (Sigma-Aldrich, Germany) and anaerobically cultured at 37 °C for 48 h and 24 h, respectively. Black colonies of *Clostridium *spp*.* on SPS agar were counted. MacConkey agar (Difco Laboratories, Detroit, MI) and BSM agar (Sigma-Aldrich, Germany) were used to cultivate *E. coli* and *Bifidobacterium *spp*.* respectively, and incubated at 37 °C for 24 h. All microbiological analyses were performed in triplicate, and average values were used for statistical analyses and results were expressed in colony-forming units (Log_10_ cfu/g of ileal content).

### RNA extraction and gene expression

The procedure of RNA extraction and gene expression was previously explained^26^. In summary, total RNA was extracted from chicken jejunum sampled on day 22 using the total RNA extraction kit (Pars Tous, Iran) following the manufacturer’s instructions. The purity and quality of extracted RNA were evaluated using an Epoch microplate spectrophotometer (BioTek, USA) based on 260/230 and 260/280 wavelength ratios, respectively. Genomic DNA was removed using DNase I (Thermo Fisher Scientific, Austin, TX, USA). The complementary DNA (cDNA) was synthesized from 1 µg of total RNA using the Easy cDNA synthesis kit (Pars Tous, Iran) following the manufacturer’s protocol.

Gene expression of two references (GAPDH and β-actin) and five targets (Interleukin-1 [IL-1], IL-6, mucin2 [MUC2], Claudin-1 [CLDN1], and Occludin [OCLN]) genes were determined by quantitative real-time PCR (qPCR) based on MIQE guidelines^27^. Each reaction was performed in a total volume of 20 μl in duplicate using an ABI 7300 system (Applied Biosystems, Foster City, CA) and 2 × SYBR Green Real Time-PCR master mix (Pars Tous, Iran). Primer details are shown in Table 2. All primers were designed according to MIQE criteria^27^ regarding amplification length and intron spanning. All efficiencies were between 90 and 110%, and calculated R^2^ was 0.99 for all reactions. The method 2^−ΔΔCt Ct^ was used to calculate relative gene expression in relation to the reference genes^28^ (GAPDH and β-actin).

**Table 2:** Sequences of primer pairs used for amplification of target and reference genes.^1^

Gene^2^	Strand	Sequence (5´ → 3´)	Ta	Product size (bp)	GenBank Accession No
ANXA1	Forward	CTGCCTGACTGCCCTTGTGA	63	98	NM_206906.1
	Reverse	GTTTGTGTCGTGTTCCACTCCC			
TRAF3	Forward	CTGAGAAAAGATTTGCCAGACCA	63	101	XM_421378
	Reverse	CATGAAACCATGACACACGGG			
MUC2	Forward	ATGCGATGTTAACACAGGACTC	60	110	BX930545
	Reverse	GTGGAGCACAGCAGACTTTG			
CLDN1	Forward	CATACTCCTGGGTCTGGTTGGT	60	100	NM_001013611.2
	Reverse	GACAGCCATCCGCATCTTCT			
OCLDN	Forward	CGCAGTCCAGCGGTTACTA	58	178	NM_205128.1
	Reverse	AGGATGACGATGAGGAACCCA			
GAPDH	Forward	TTGTCTCCTGTGACTTCAATGGTG	63	128	NM_204305
	Reverse	ACGGTTGCTGTATCCAAACTCAT			
β-Actin	Forward	CCTGGCACCTAGCACAATGAA	63	175	NM_205518.1
	Reverse	GGTTTAGAAGCATTTGCGGTG			

^1^For each gene the primer sequence for forward and reverse (5´ → 3´), the product size (bp), and the annealing temperature (Ta) in °C are shown.

^2^ANXA1, annexin A1; TRAF3, tumor necrosis factor receptor associated factor 3; MUC2, mucin2; CLDN1, claudin1; OCLDN, occludin; GAPDH, Glyceraldehyde 3-phosphate dehydrogenase.

### Statistical analysis

Data were statistically analyzed in a completely randomized design by ANOVA using the General Linear Model (GLM) procedure of SAS (SAS Inst., Inc., Cary, NC). Tukey’s test was used to compare differences among means of treatments, and *P* values < 0.05 were considered to be significant.

## Results

### Lesion score and mortality

Table 3 shows the effects of experimental treatments on NE-inducing lesion scores in different segments of the intestine and mortality rate of broiler chickens. The results showed that none of the additives could rehabilitate the NE-inducing lesions in the intestine compared to NC. While the highest lesion scores in the intestine of PC group showed that NE was efficiently induced in broilers, AMP decreased (*P* < 0.05) lesions in duodenum, jejunum, and ileum of broilers compared to the challenged group.

**Table 3:** Effects of treatments on necrotic enteritis lesion scores (d 22) and mortality (d 16–22) in broiler chickens.

Treatments	Lesion score			Mortality^2^ (%)
	Duodenum	Jejunum	Ileum	
NC^1^	0.000^c^	0.000^c^	0.000^c^	0.000^c^
PC	0.187^a^	1.131^a^	0.944^a^	8.17^a^
AMP	0.093^b^	0.769^b^	0.621^b^	2.53^bc^
Antibiotic	0.148^ab^	0.955^ab^	0.783^ab^	3.44^b^
SEM^3^	0.119	0.285	0.373	0.819
*P*-value	0.034	0.001	0.013	0.001

^a–c^Values within a column with different letters differ significantly (*P* < 0.05).

^1^NC: negative control group received corn-soybean meal diet without challenge and additives; PC: positive control group received NC diet experimentally challenged with necrotic enteritis; AMP: PC received group supplemented with 20 mg antimicrobial peptide/ kg diet; Antibiotic: PC received group supplemented with 45 mg antibiotic (bacitracin methylene disalicylate)/kg diet.

^2^Only mortalities shown necrotic enteritis signs.

^3^SEM: standard error of means (results are given as means (n = 12) for each treatment).

Inducing NE in broilers increased (*P* < 0.05) mortality, while birds fed with peptide showed lower (*P* < 0.05) mortality rate compared to PC group and interestingly had similar results to NC group.

### Broiler performance

Table 4 represents the effects of experimental diets on growth performance of broilers. While the antibiotic improved (*P* < 0.05) birds’ ADG at first 10 days of age, peptide showed the best FCR at the end of starter period when no challenge was induced yet. At d 22, NE challenge decreased (*P* < 0.05) ADG and increased (*P* < 0.05) feed intake, which devastated (*P* < 0.05) FCR in broilers. In the latter phase, peptide showed similar effects to NC group, while improved (*P* < 0.05) performance indices compared to PC group.

**Table 4:** Effects of treatments on growth performance of broiler chickens from 0 to 22 days of age.

Treatments	ADG^2^ (g)			ADFI (g)			FCR (g/g)		
	0–10	11–22	0–22	0–10	11–22	0–22	0–10	11–22	0–22
NC^1^	26.25^b^	58.95^b^	85.20^b^	24.93^a^	74.79^b^	99.37^b^	0.950^a^	1.269^b^	1.170^b^
PC	26.15^b^	55.91^c^	82.06^c^	23.77^ab^	88.18^a^	111.95^a^	0.909^a^	1.578^a^	1.364^a^
AMP	27.05^ab^	60.29^ab^	87.34^ab^	22.61^b^	72.23^b^	94.85^b^	0.836^b^	1.199^b^	1.086^c^
Antibiotic	27.78^a^	61.94^a^	89.72^a^	24.64^a^	75.26^b^	99.90^b^	0.888^ab^	1.215^b^	1.113^bc^
SEM^3^	0.318	0.446	0.644	0.422	1.401	1.577	0.0164	0.0257	0.0181
*P *value	0.007	0.001	0.001	0.005	0.001	0.001	0.001	0.001	0.001

^a–c^Values within a column with different letters differ significantly (*P* < 0.05).

^1^NC: negative control group received corn-soybean meal diet without challenge and additives; PC: positive control group received NC diet experimentally challenged with necrotic enteritis; AMP: PC received group supplemented with 20 mg antimicrobial peptide/ kg diet; Antibiotic: PC received group supplemented with 45 mg antibiotic (bacitracin methylene disalicylate)/kg diet.

^2^ADG: average daily gain; ADFI: average daily feed intake; FCR: feed conversion ratio.

^3^SEM: standard error of means (results are given as means of 6 pens of 15 birds/treatment).

### Jejunal villi morphology

The effects of treatments on jejunal morphology are shown in Table 5. On day 10, experimental diets had no significant effects on the morphometry of the intestine. NE challenge significantly demolished villi structure and morphology, while AMP enhanced (*P* < 0.05) villus height, width, and surface area (VSA) compared to PC group and had a similar effect to NC group at 22 days of age. Experimental diets had no significant effects on CD and VH/CD at d 22. Although the antibiotic improved (*P* < 0.05) villi morphology compared to NE-challenged birds, it could not restore villi characteristics to those of NC.

**Table 5:** Effects of treatments on villi morphology (µm) in the jejunum of broiler chickens at 10 and 22 days of age.

Treatment	Day 10					Day 22				
	VH^2^	VW	CD	VH/CD	VSA (µm^2^)	VH	VW	CD	VH/CD	VSA (µm^2^)
NC^1^	621	188	144	3.29	367.7	1175^a^	186^a^	187	5.69	688.8^a^
PC	592	192	125	3.09	356.6	827^c^	153^b^	201	5.04	396.9^c^
AMP	681	194	138	3.52	414.2	1140^ab^	187^a^	171	6.06	671.5^a^
Antibiotic	641	197	121	3.26	396.9	1017^b^	174^a^	180	6.50	557.0^b^
SEM^3^	26.4	3.5	13.7	0.164	16.42	35.4	5.1	22.8	0.632	25.78
*P* value	0.167	0.401	0.610	0.348	0.102	0.001	0.001	0.816	0.447	0.001

^a–c^Values within a column with different letters differ significantly (*P* < 0.05).

^1^NC: negative control group received corn-soybean meal diet without challenge and additives; PC: positive control group received NC diet experimentally challenged with necrotic enteritis; AMP: PC received group supplemented with 20 mg antimicrobial peptide/ kg diet; Antibiotic: PC received group supplemented with 45 mg antibiotic (bacitracin methylene disalicylate)/kg diet.

^2^VH: villus height; VW: villus width; CD: crypt depth; VH/CD: the ratio of VH to CD; VSA: villus surface area.

^3^SEM: standard error of means [results are given as means (n = 12) for each treatment].

### Bacterial colonization

Table 6 summarizes the effects of experimental diets on ileal bacterial populations. At d 10, the antibiotic decreased (*P* < 0.05) the population of all bacteria compared to other groups. NE challenge increased (*P* < 0.05) the count of *E. coli* and expectedly *Clostridium *spp. and also decreased (*P* < 0.05) the number of *Lactobacillus *spp. and *Bifidobacterium *spp. in the ileum of birds. In the same period, the antibiotic had the lowest (*P* < 0.05) population of all cultured ileal bacteria compared to both PC and NC groups. Interestingly, AMP had similar effects to NC group, while it increased (*P* < 0.05) the population of *Lactobacillus *spp*.* and *Bifidobacterium *spp*.* and also decreased (*P* < 0.05) the colonization of *E. coli* and *Clostridium *spp*.* in the ileum of chickens compared to the challenged birds at 22 days of age.

**Table 6:** Effects of treatments on ileal microflora (log_10_ CFU g^-1^) in broilers at 10 and 22 days of age.

Treatments	Day 10				Day 22			
	*E. coli*	*Lactobacillus *spp*.*	*Bifidobacterium *spp*.*	*Clostridium *spp*.*	*E. coli*	*Lactobacillus *spp*.*	*Bifidobacterium *spp*.*	*Clostridium *spp*.*
NC^1^	3.03^a^	5.69^a^	6.17^a^	1.62^a^	4.09^b^	7.36^a^	6.41^a^	2.74^c^
PC	3.35^a^	5.39^a^	6.49^a^	1.66^a^	5.11^a^	5.21^b^	4.32^b^	5.45^a^
AMP	2.31^ab^	5.31^a^	6.47^a^	1.48^ab^	3.72^bc^	6.69^a^	5.86^a^	4.68^b^
Antibiotic	1.87^b^	3.83^b^	4.73^b^	1.31^b^	2.80^c^	5.37^b^	4.54^b^	4.38^b^
SEM^2^	0.263	0.267	0.328	0.062	0.233	0.311	0.241	0.074
*P* value	0.007	0.002	0.007	0.008	0.001	0.009	0.001	0.001

^a–c^Values within a column with different letters differ significantly (*P* < 0.05).

^1^NC: negative control group received corn-soybean meal diet without challenge and additives; PC: positive control group received NC diet experimentally challenged with necrotic enteritis; AMP: PC received group supplemented with 20 mg antimicrobial peptide/ kg diet; Antibiotic: PC received group supplemented with 45 mg antibiotic (bacitracin methylene disalicylate)/ kg diet.

^2^SEM: standard error of means (results are given as means (n = 12) for each treatment).

### Gene expression of immune cells and tight junctional proteins

**Figure 1:**
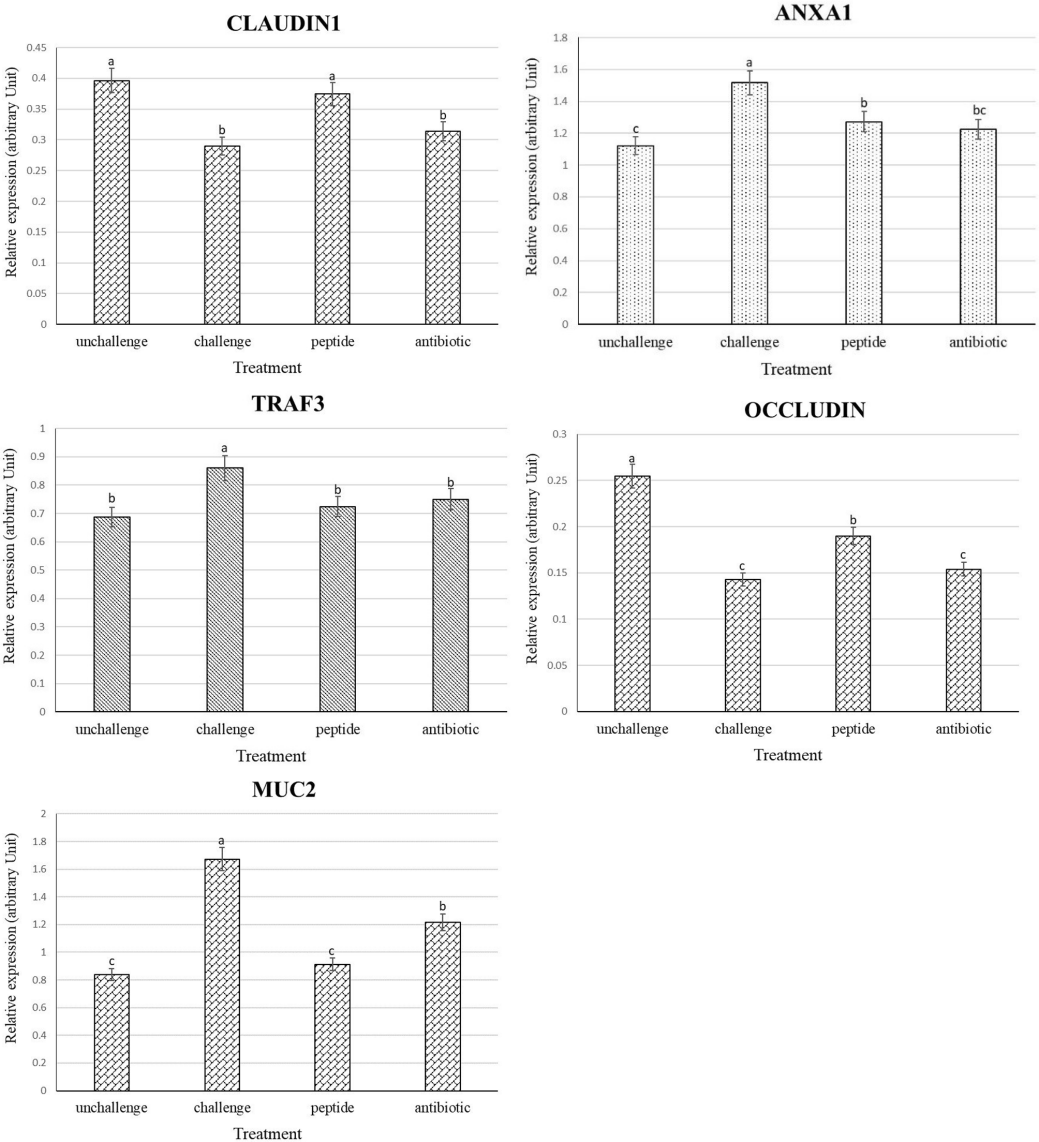
Effects of treatments on the expression of different genes in the jejunum of broiler chickens on day 22. Samples were analyzed using qPCR, and GAPDH and β-actin were used as the reference genes. Abbreviations as follows: ANXA1, annexin A1; TRAF3, tumor necrosis factor receptor associated factor 3; MUC2, mucin2; unchallenge, control birds received a corn-soybean meal basal diet without AMPs, antibiotic and necrotic enteritis (NE) challenge; challenge, control birds challenged with NE; peptide, birds challenged with NE and supplemented with 20 mg cLFchimera/kg diet; Antibiotic, birds challenged with NE and supplemented with 45 mg antibiotic (bacitracin methylene disalicylate)/kg diet; The letters on the bar mean show significant difference (*P* < 0.05).

The effects of treatments on the expression level of immune and tight junction genes are presented in Fig. 1. While NE challenge increased (*P* < 0.05) TRAF3 and ANXA1 transcripts, adding the antibiotic and AMP to the diet reduced (*P* < 0.05) the expression of immune genes compared to PC group and the antibiotic had similar effects to those of non-challenged birds.

While NE challenge increased the expression of MUC2 in the jejunum of birds, AMP decreased (*P* < 0.05) the level of MUC2 trasncripts compared to PC group and had similar levels to NC group. Birds challenged with NE had the lowest expression levels of CLDN1 and OCLN genes in their jejunum. AMP increased (*P* < 0.05) expression patterns of CLDN1 in the jejunum of birds compared to PC group and had similar effects to those of non-challenged birds. Although birds fed with AMP had higher (*P* < 0.05) OCLN mRNA in the jejunum compared to PC group, this group had lower (*P* < 0.05) expression of this transcript compared to NC group. The antibiotic did not affect the level of tight junctions transcripts in the jejunum of NE challenged birds compared to both NC and PC groups.

## Discussion

Necrotic enteritis is still a global concern with drastic losses in poultry farms, mainly due to retarded growth performance, increased mortality, and veterinary costs^23^. The outbreak of disease and consequently economic losses have been more prominent in the post-antibiotic era^23^. Recently, research focus has shifted to AMPs due to their beneficial roles on health attributes and their prophylactic effects against pathogenic invasion^12,13,17^. Therefore, the principal objective of the current study was to investigate the effects of antimicrobial peptide, cLFchimera, on various productive and health parameters in chickens challenged with NE.

Results of the current study showed that AMP decreased gut lesions and mortality induced by NE. Additional benefits include also improved growth attributes in challenged chickens similar to birds fed antibiotic, confirming the results of previous studies^9,10^. While most of the previous researches have studied the effects of AMPs in chickens in normal conditions, Hu et al.^29^ demonstrated that supplementing broilers diet with AMP improved their weight gain and FCR under heat stress. In another challenge study, Wu et al.^30^ inoculated weanling pigs with *E. coli* and supplemented the diet with AMP. The autors reported that AMP reduced the incidence of diarrhea and improved weight gain and FCR compared to the challenged group, which is similar to the present findings regarding the reduction in gut lesion and improvement of performance. Previous studies attributed the beneficial effects of AMPs on growth performance of chickens to their fundamental roles in maintaining microbial balance in the gut and consequently improvement in the intestinal morphometry^9,10^.

It has been well-documented that the villi play the critical roles in absorbing nutrients from the intestinal tract; consequently the morphometry of these villi can drastically affect the host’s performance and health^31^. The present study confirmed, AMP significantly improved the morphometry of villi in the jejunum of challenged chickens, similar to that of NC group^9,10^. It has been reported that AMPs extracted from pig intestine^32^ and rabbit sacculus rotundus^33^ enhanced jejunal villi characteristics in broiler chickens, which is in line with the present results. Generally, the critical strategy in maintaining villi structure in an infectious disease like NE is the elimination or minimization of the pathogens through provision of antimicrobial additives and manipulation of the intestinal microbiome^34,35^. Previous studies showed that the antibiotic and AMPs could improve villi morphology and nutrient absorption and consequently increase growth performance in chickens under disease conditions by manipulating the intestinal microflora^9,10,17^.

The intestinal commensal microbiome interacts with the host through different processes, including nutrients absorption, villi morphology, intestinal pH, and mucosal immunity^36,37^. In the current study, the supplementation of the antibiotic reduced the colonization of all bacteria, while AMP significantly enhanced the microflora balance in the ileum. In agreement with the present study, Tang et al.^7^ and Ohh et al.^38^ reported that AMPs significantly enhanced the microflora balance in the ileum of piglets and broilers, respectively. The antimicrobial action of Bacitracin Methylene Disalicylate (BMD) involves blocking the bacterial ribosome subunits and subsequently impeding protein synthesis, which finally reduces the colonization of the microbial community in the intestine^39^. Unfortunately, this antibiotic does not differentiate between commensal vs. pathogenic bacteria and may perturb microbial balance in the intestine and deprive the host of benefits of microbes’ roles and products^39,40^. There is no consensus on the mechanism by which AMPs influence bacterial colonization in the intestine, while two direct and indirect mechanisms have been proposed based on the physiological properties of peptides. The direct antimicrobial effect of AMPs has been attributed to different surface charges of peptides and pathogens^41^. In other words, AMPs possess positive charges contributing to electrostatically adhere to negatively charged bacterial membranes^41,42^. This attachment can either destroy the bacterial membranes through physical disruption or penetrate the bacterial cytoplasm without exerting any damage to the lipid layer^41,43^. Imported AMPs may interfere with intracellular signaling pathways like nucleic acids synthesis, enzyme activity, and protein biosynthesis^42,43^. In the indirect mode, AMPs might manipulate the microbial community of the intestine in favor of the colonization of beneficial bacteria (e.g. *Lactobacillus *spp. and *Bifidobacterium *spp.) and enhance the host health through various physiological mechanisms (e.g. competitive exclusion, secretion of short-chain fatty acids, activation of intestinal immune system, etc.)^42^. Previous findings suggested that cLF36 could attach to the bacterial membrane through electrostatic interactions and physically disrupt bacterial bilayer membranes^12,13,16^. In line with the previous reports^44^, the current results demonstrate that AMP can selectively prevent the bacterial growth in the intestine of NE challenged chickens, which may prove the competitive advantage of cLFchimera compared to antibiotics. Furthermore, previous research reported that the antimicrobial activities of AMPs against pathogens in the intestine might alert the host immune system to fight against invading agents^45,46^.

Mucosal immunity plays an important role in host defense against pathogens^47^. In broiler chickens, when Toll-like receptor (TLR)4 engages to microbe-associated molecular patterns (MAMP), it transmits the information to the cytoplasm of the phagocytes, which in turn leads to the expression of cytokines^48,49^. Different research groups have reported the controversial results regarding the effects of *C. perfringens* challenge on gene expression of TLR4 in the intestine of broiler chickens. For instance, while some researchers reported that *C. perfringens* upregulated the TLR4 gene expression in the intestine of chickens^50,51^, other investigators reported no apparent alteration of the TLR4 gene expression in *C. perfringens* challenged chickens^52^. Therefore, in the current study, we decided to analyze the gene expression of TRAF3, which is one step ahead of TLR4 activation to overcome the possible interference of other immune cells^53^. TRAF3 is a cytoplasmic protein that controls signal transduction from different receptor families, especially TLRs^53^. Following the activation of TLR4 with pathogen attachment, TRAF3 is recruited into signaling complexes, and its activation increases vital pro-inflammatory cytokines production^54,55^. Results of the present study showed that NE upregulated the expression of TRAF3 transcript in the jejunum of chickens, while the antibiotic and AMP significantly decreased the gene expression of this cytokine in the challenged birds, which is consistent with the results of previous studies^54,56^.

On the other hand, excessive and long-term production of pro-inflammatory cytokines might result in the gut damage and high energy demand^57^. To prevent the adverse effects of extra pro-inflammatory cells, pro-resolving mediators such as ANXA1 are released into the epithelial environment to orchestrate clearance of inflammation and restoration of mucosal homeostasis^58,59^. ANXA1 is a 37 kDa protein expressed in the apical and lateral plasma membrane in the intestinal enterocytes that facilitates the resolution of inflammation and repair^60^. In the current study, *C. perfringens* upregulated the expression of ANXA1 mRNA in the jejunum of PC group, which is in agreement with previous report^54,61^, while the antibiotic and AMP significantly decreased the expression of this cytokine, which is firstly reported herein. While there is no well-documented evidence to explain the results of cytokines expression, it could be inferred that antimicrobial activities of the antibiotic and AMP in the current study resulted in the reduction of invading pathogens (based on abovementioned microbial results) in the intestine of PC birds and possibly downregulating the expression of both pro- and anti-inflammatory (i.e. TRAF3 and ANXA1, respectively) cytokine-producing cells.

The epithelial barrier consists of tight junction proteins forming the primary lines of defence against a wide range of stimuli from feed allergens to commensal and pathogenic bacteria^62,63^. The disruption of these proteins may result in increasing the intestinal permeability to luminal pathogens^62,63^. Previous studies showed that *C. perfringens* might attach to the junctional proteins to form gaps between the epithelial cells and disrupt the intestinal integrity^63,64^. In the present study, NE challenge reduced the jejunal expression of OCLN and CLDN1 transcripts, which is in agreement with previous studies^64,65^. Previous studies showed that tight junction proteins, especially CLDN1 and OCLN, have a specific region (i.e. ECS2) containing a toxin-binding motif, NP (V/L)(V/L)(P/A), that is responsible for binding to *C. perfringens*^63,66^. Following attachment to junctional proteins, *C. perfringens* could digest these proteins^67^ and open the intracellular connection between adjacent epithelial cells resulting in more penetration of pathogens to deeper layers of lamina propria and transmitting to other organs^63,68^. AMP significantly upregulated the expression of these genes in PC group and the antibiotic had no significant effect on the gene expression of junctional proteins. In agreement with the current findings, previous reports demonstrated that AMPs could increase the expression of junctional proteins in different challenge conditions^17,69^. While no exact mechanism has been recognized, there are two suggested theories for the inhibitory effects of AMPs on *C. perfringens* regarding junctional proteins. In the first theory, it has been suggested that AMPs could directly switch on the expression of regulatory proteins (i.e. Rho family) in the intestine of challenged mice, upregulating the expression of tight junction proteins and ameliorating leaky gut^69,70^. The second theory attributed the beneficial effects of AMPs on tight junctions to their indirect roles in manipulating microflora populations in the intestine. In detail, previous studies showed that the intestinal commensal bacteria like Bifidobacteria and Lactobacilli secrete butyric acid that regulates epithelial O_2_ consumption and stabilization of hypoxia-inducible factor. This transcription factor protecting the epithelial barrier against pathogens, resulting in higher expression of junctional proteins^71,72^. Therefore, it can be hypothesized that AMP in the current study upregulated the expression of junctional proteins by both reducing the number of *C. perfringens* and inhibiting protein disruption by bacterial toxins. Surprisingly, the antibiotic did not change the expression of CLDN1 and OCLN transcripts in the jejunum of chickens, while it could be expected that the antibiotic upregulated the junctional proteins due to the antibacterial nature of antibiotics. In line with the current results, Yi et al.^69^ reported that antibiotics might not affect the gene expression of junctional proteins of the epithelial cells after pathogen removal, maybe because of controlling the microbial balance in the intestine.

Along with junctional proteins, the luminal mucus layer comprising of mucins plays a defensive role against invasive pathogens^73^. MUC2 widely expresses in goblet cells and secretes into the intestinal lumen to stabilize mucosal layer^73,74^. Any damage to the mucosal layer stimulates the expression of MUC2 to secrete more mucin and prevent further destruction^74,75^. In the current study, NE significantly increased the gene expression of MUC2 in the jejunum, which is in agreement with the results of previous studies^76,77^. On the other hand, the antibiotic and AMP significantly downregulated the level of this transcript, while the results for AMP was similar to those of NC group. According to the bacterial results in the present study, it could be inferred that the inhibitory effects of AMP on the population of *C. perfringens* and *E. coli* might reduce the colonization of these bacteria in the intestine, decrease the destruction of the mucosal layer, and subsequently lessen the expression of MUC2 transcript. The exact mechanism of these effects has not yet been revealed.

In conclusion, results of the current study propose that cLFchimera, an antimicrobial peptide originating from camel milk, could reduce mortality and attenuate NE-induced lesions in broilers. Beneficial consequences of AMP use include better growth performance and recovery of villi morphology in the jejunum of NE-imposed chickens. Further, supplementing NE challenged birds with cLFchimera restored the ileal microflora and consequently regulated the expression of cytokines, MUC2, and tight junctional proteins. Therefore, according to the desired results obtained in the present study, cLFchimera can be nominated as a candidate for replacing growth-promoting antibiotics against NE in chickens, while further studies may find other favourable effects of this AMP.

## Data Availability

All generated and analysed data in the current study are included in this article, and also cited data were included in the reference list.
